# Carbon-Based Polymer Nanocomposite for High-Performance Energy Storage Applications

**DOI:** 10.3390/polym12030505

**Published:** 2020-02-26

**Authors:** Samarjeet Singh Siwal, Qibo Zhang, Nishu Devi, Vijay Kumar Thakur

**Affiliations:** 1 Key Laboratory of Ionic Liquids Metallurgy, Faculty of Metallurgical and Energy Engineering, Kunming University of Science and Technology, Kunming 650093, China; samarjeet6j1@gmail.com; 2 State Key Laboratory of Complex Nonferrous Metal Resources Cleaning Utilization in Yunnan Province, Kunming 650093, China; 3 Department of Chemistry, University of Johannesburg, P.O. Box: 524, Auckland Park 2006, South Africa; 4 Enhanced Composites and Structures Center, School of Aerospace, Transport and Manufacturing, Cranfield University, Bedfordshire MK43 0AL, UK; 5 Department of Mechanical Engineering, School of Engineering, Shiv Nadar University, Uttar Pradesh 201314, India

**Keywords:** carbon-based polymer nanocomposite, energy storage, fuel cell, electrochemical devices

## Abstract

In recent years, numerous discoveries and investigations have been remarked for the development of carbon-based polymer nanocomposites. Carbon-based materials and their composites hold encouraging employment in a broad array of fields, for example, energy storage devices, fuel cells, membranes sensors, actuators, and electromagnetic shielding. Carbon and its derivatives exhibit some remarkable features such as high conductivity, high surface area, excellent chemical endurance, and good mechanical durability. On the other hand, characteristics such as docility, lower price, and high environmental resistance are some of the unique properties of conducting polymers (CPs). To enhance the properties and performance, polymeric electrode materials can be modified suitably by metal oxides and carbon materials resulting in a composite that helps in the collection and accumulation of charges due to large surface area. The carbon-polymer nanocomposites assist in overcoming the difficulties arising in achieving the high performance of polymeric compounds and deliver high-performance composites that can be used in electrochemical energy storage devices. Carbon-based polymer nanocomposites have both advantages and disadvantages, so in this review, attempts are made to understand their synergistic behavior and resulting performance. The three electrochemical energy storage systems and the type of electrode materials used for them have been studied here in this article and some aspects for example morphology, exterior area, temperature, and approaches have been observed to influence the activity of electrochemical methods. This review article evaluates and compiles reported data to present a significant and extensive summary of the state of the art.

## 1. Introduction

Renewable sources—for example, solar and wind energy—can satisfy the world’s power needs, but substitutes for petroleum-derived substances demand a root of carbon fragments [1]. As renewable sources are not spontaneous sources of energy, therefore, storage of that energy generated from renewable sources is a prerequisite for its later use. Also, the innovation of new and compact portable electronic devices has drawn the attention towards efficient energy storage. In the prior two eras, transportable tools/portable devices such as smartphones, laptops, smart health devices, have reduced suggestively in volume, and besides their abilities, storage potentials continue to rise intensely. Not surprisingly, the energy markets of these tools are rather abundant, which has led to a massive improvement in the level of study towards high-performance applications. In current times, millions of dollars have been funded for the investigations that are focused on the evolution of more effective energy storage systems, including fuel cells [2,3,4,5,6,7,8], supercapacitors [9], and batteries [10]. Among these applications, battery technology has been one of the most established systems for energy storage as it is useful for high energy requirements owing to their high energy capabilities. Though, despite the dramatic performance with time, there is yet notable room for the development.

Carbon-based polymer nanocomposites (CPNCs) have various applications in the energy accumulation, energy storage, packing, aerospace, and automotive areas [11,12]. The important characteristics of these nanostructured substances are the comfort of processing, configuration adaptability, lightweight, and flexibility to requirements. For energy storage, fuel cell and supercapacitors are supposed to be crucial components in updating the prospect of renewable energy schemes [13,14]. The demand for high energy and power density devices at a low-cost leads to the discovery of novel nanocomposite materials for automotive and electric energy storage applications. Insulating polymers loaded by high-aspect-ratio conductive nanofillers—for example, carbon nanotube (CNT) [15,16] as well as graphene nanoplatelets (GNP) [17,18]—have been proved to be potential dielectric ingredients [19,20]. Scattered nanocomposites within an insulating pattern performed as nanocapacitor electrodes that drive significant improvement in space charge polarization [21,22]. The nanofiller is assumed to become highly conductive to improve the interfacial polarization on the nanofiller/polymer boundary and high exterior area to develop the interfacial polarization density. As a polymer nanocomposite, the polymer matrix, nanofiller, and polymer/nanofiller interface may be polarized. It is observed that this polarization in nanocomposites is plausible, particularly at quite large frequencies, though the polarization of the conductive nanofiller and the interfacial region may happen frequencies under 1 MHz [23,24].

The energy storage methods require unique, paramount, and authentic approaches towards the storage of electric power by alternate renewable origins for assuring appropriate and dependable devices that can store a sufficient quantity of energy and later can be used for transport, electronic gadgets, electric-powered carriers, and distinct purposes. Electrochemical energy storage operations comprise supercapacitors, various kinds of batteries, and fuel cells [25]. Another energy storage method, for example, thermal, compressed air, also flywheel energy aggregate nonelectrochemical storage operations [26,27,28]. The electrochemical storage methods usually contain the probes, electrolyte media, and power receiver. These ingredients are built from carbon-based nanomaterials, CPs, metal oxides (MO_x_), as well as conducting substances. These substances are not well established and there are yet numerous hurdles that need to designate and overcome for the further extensive commercialization of the energy storage applications. Recently, excellent new substances called CPNCs have been examined for electrochemical applications [29].

In the present article, the formerly published data is explained by giving a rationalized review of the state of the system and setting up a wide series of electrochemical energy storage (EES) technologies. EES technology helps in justifying the inconsistency of renewable resources, energy production from them, and storage of that energy. This article includes the reflection on CPNCs for energy storage devices and their high-performance applications. In the remainder of this review article, we briefly presented the characteristics and applications of several kinds of carbon-based polymer nanocomposites towards its use as ingredients of high-performance energy storage substances. An up-to-date explanation of the most advanced progress in the growth of CPNC ingredients intended for their uses as an electrode material probe and electrolyte substantial for fuel cell, supercapacitor (SC), and battery.

## 2. Electrochemical Energy Storage Applications 

**Figure 1:**
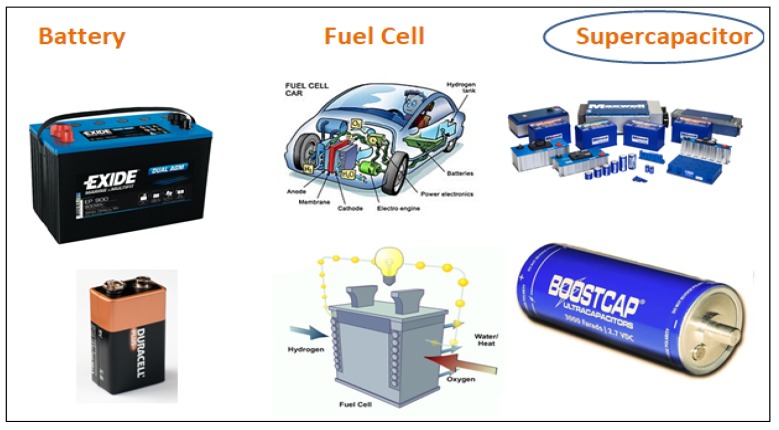
Types of electrochemical energy storage devices.

One of the paramount scientific and societal tasks in front of us is to provide secure energy for future generations. The prerequisite for storing energy is giving us ideas to explore more and more inexpensive techniques for energy storage. Although researchers and scientists are heading towards renewable sources of energy but to store that renewable energy for future use, we require energy storage devices. One provision is storing energy electrochemically using electrochemical energy storage devices like fuel cells, batteries, and supercapacitors (Figure 1) having a different mechanism of energy storage but have electrochemical resemblances. 

Energy exists in various forms in nature like radiation, chemical, gravitational, potential, electricity, latent heat, kinetic energy, etc. Energy storage is the capture of produced energy so that it can be used later. Battery and supercapacitors are most commonly used energy storage devices, but fuel cell does not store energy instead produces energy directly from the fuel and oxygen via electrochemical reactions in the cell. The basic operating mechanism and a brief background of these three electrochemical systems is explained in the next few sections.

### 2.1. Fuel Cells

**Figure 2:**
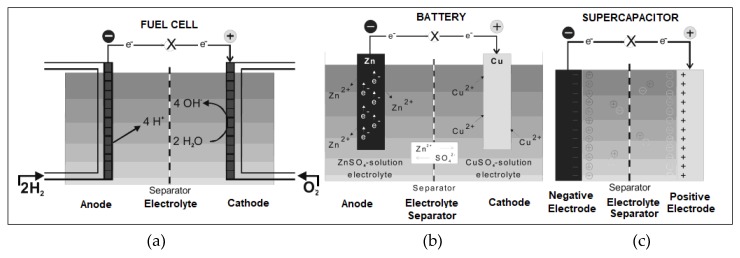
Representation of a fuel cell (**a**) shows the constant source of ingredients and redox reactions in the cell, a battery (**b**) shows the salient features of battery operation and a supercapacitor (**c**) illustrating the energy storage at probe-conducting solution interface. Reproduced with permission from [30].

The first fuel cell (FC) was invented in 1838 by Sir William Grove. The first commercial use of fuel cells was in NASA space programs. FC is electrochemical equipment that harvests electricity directly from fuel instead of accumulating. In addition, they require combustible fuels in stock and a continuous supply of oxygen from the atmosphere. It renovates the chemical energy of the fuel directly into electricity by redox reactions in the cell. They are different from the battery in the location of energy storing and alteration. Fuel cells are open systems, in case of fuel cell, fuels that provide energy are located outside, e.g., oxygen in the air and hydrogen outside in the fuel storage tank which are supplied continuously to the reaction chamber (Figure 2, fuels are supplied from outside into the reaction chamber) while battery is a closed system through the positive and negative electrode as the charge transfer intermediate [30]. Fuel cells have very high energy density even higher than batteries but the lowest power density due to which they are not widely employed towards energy storage and renovation. FCs are still in the developing phase and in search of an effective application that can penetrate the energy market but still be competitive against batteries and supercapacitors.

### 2.2. Battery

Battery stores energy via electrochemical reactions. In comparison to fuel cells and supercapacitors, batteries have established the energy storage market till now and most commonly used in mobile phones, laptops, automobiles, homes, offices, portable, mobile, and stationary applications. Batteries vary in size and capacity from an aspirin tablet 1–10 mAh used under air range aids to large buildings with 40 MWh towards energy storage and backup use [30]. Various battery systems like lead–acid, Ni–Cd, Ni–Zn, rechargeable alkaline, Li-ion, and Li-ion polymers are in use.

**Figure 3:**
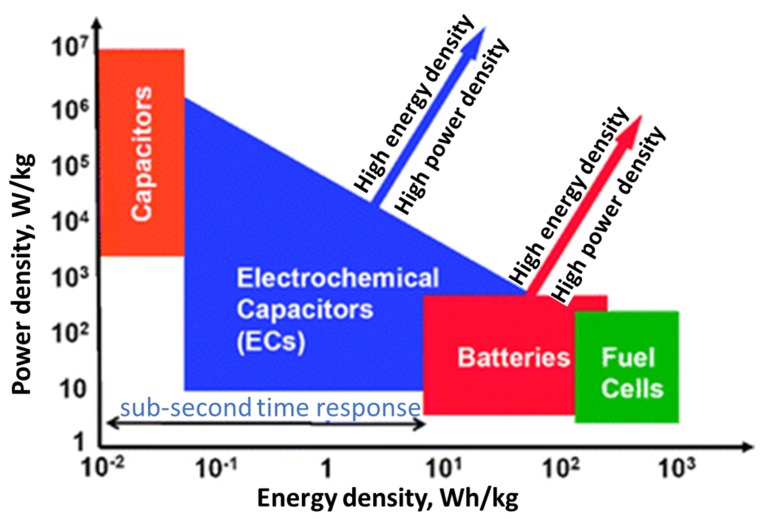
Ragone plot representing the distribution of ED and PD of electrochemical energy storage devices. Reproduced with permission from [31].

A simple Ragone plot (Figure 3) shows that FCs are high energy structures whereas SCs are high energy arrangements, batteries are somewhere in between the two but overlapping a part of SC and fuel cells (FCs) [31]. That is why SC and FCs can replace some battery applications. Utilizing high ED of batteries and high PD of SC, a hybrid system [32] may be used to harvest essential properties of both the systems which can be used in practical applications [33]. This combination of both systems can meet the requirements of future power applications as hybrid systems [32].

### 2.3. Supercapacitor 

Renewable energy schemes have noticeably been painstaking as an influential armament to employ in the environmental revolution and combat global warming [34,35,36]. In recent years, wind and solar energy have seen unexpected development with enormous cost drops, which has made them more attractive relative to fossil fuels. Though, owing to the worries in power production and demand, energy storage strategies have dynamic appeal in making flexible and dependable energy structures.

Supercapacitors or ultracapacitors having capacitance much higher than usual capacitors are electrochemical energy storage tools which store energy either electrochemically at the exterior of the electrode–electrolyte interface (Figure 2), also known as an electrochemical double-layer capacitor (EDLC) or by redox responses occurring on the surface of electrode recognized as pseudocapacitors. In 1950, General Electric Engineers started experiments using porous carbon electrodes. H. Becker established a low voltage capacitor using the same porous carbon electrodes in 1957 [37]. Recently, Li-ion capacitors came into knowledge and still researchers are aiming to enhance or upgrade specific energy, specific power, cycle stability, and trying to diminish the cost to replace a battery in the energy market [38]. SCs are EDLC, pseudocapacitors, and hybrid capacitors that are pivotal in the mechanism of charge storage and ingredients employed [39].

However, still, SCs are facing material stability challenges, and we need research these issues, so that supercapacitors can also be used as current devices which require high capacity for brief periods. The performance of SC depends upon many factors including electrochemical characteristics of probe material, type of electrolyte in addition to the voltage assortment. Two energy storage mechanisms, EDLC and pseudocapacitive, have been studied depending upon the type of material carbonaceous, metal oxide, or polymers [40,41]. Where two or more above stated materials are used in combination with each other to increase the performance of SC, both the EDLC and redox mechanisms take place in the system [42]. In asymmetric SCs, a combination of EDLC and pseudocapacitor in which one electrode is an electrical double layer electrode that stores energy via the adsorption/desorption of solution ions at the interface, whereas the other electrode is a faradic or pseudo capacitor electrode at which energy is stored by the transfer of electrons during redox reactions. A comparison between EDLC and pseudocapacitive mechanisms are shown in Table 1. Metal oxides, metal sulfides in a combination of carbon materials, have been used for asymmetric or hybrid SCs.

## 3. Carbon-Based Polymer Nanocomposites

Carbon nanomaterials, for example, activated carbon (AC) [43], graphene [44], CNTs [45], carbon dots (C dots) [46], and carbon aerogels (CAGs) [47] can act as potential filler for polymeric materials and help in enhancing the properties of the polymer. The dimensions of nanocomposite have been found to be less than 100 nm. Adding carbon nano-fillers to polymer background results in the high exterior to volume proportion and significantly improves the macroscopic possessions of the polymer including improved mechanical properties and high flexibility. 

### 3.1. Carbon Materials 

**Figure 4:**
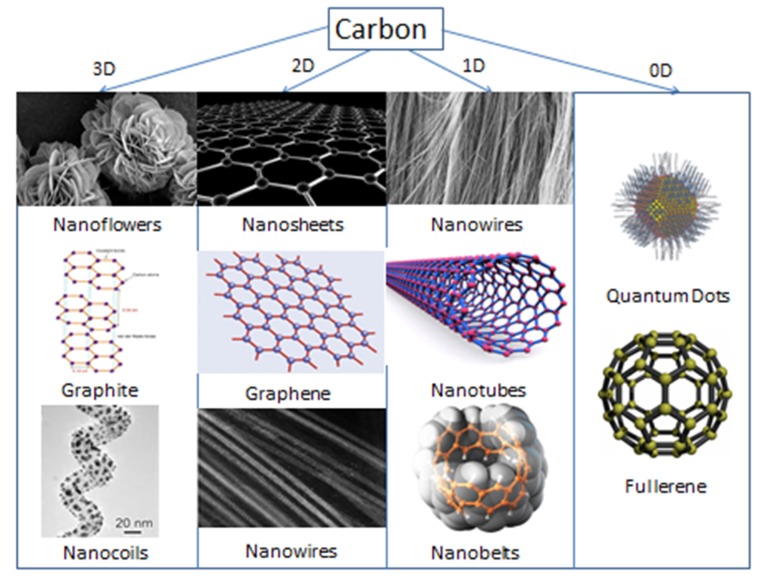
Carbon nanostructures in all three dimensions. Reproduced with permission from [48].

Carbon has been found to exist in various allotropic forms and sizes from insulator diamond to layered semiconductor graphite to fullerenes and is a versatile element of the periodic table that exhibits outstanding traits. Figure 4 shows the derived nanostructures of carbon in different dimensions (0D, 1D, 2D, and 3D) [48]. Carbon nanomaterials show exceptionally high surface areas, high electronic conductivity, amended capacitance, and reprocessing rate ability on the traditional engineering probe materials, for example, AC. Carbon-based substances, as well as AC, CNTs, along with graphene (GN), have been utilized into EDL SCs owing to their extraordinary chemical in addition to physical assets [49].

Carbon nanostructures, particularly CNTs and GN, that have been used as electrodes for supercapacitors and batteries follow the EDLC mechanism of charge storage at the electrode/solution boundary. There are some limitations of using carbon materials as electrode materials because the storage of charge is limited only by adsorption–desorption of ions at the interface of electrode/solution forming a double layer, and their aggregation during synthesis and processing results in inferior device performance. Their nanocomposites with conducting polymers have high performance and improved properties [50,51]. Carbon nanomaterials—such as CNT [52], GN [53], GN foams [54], and carbon nanofibers [45,55]—with their 3D and 2D architectures have been broadly described owing to their ideal morphological and physical possessions for energy storage applications. 

One of the most widely used carbon materials is activated carbon. The AC of high exterior area 1401 m^2^·g^−1^ by a typical pore dimension of 2.2 nm was employed for in situ growth of Bi_2_O_3_ nanoparticles and used as an asymmetric supercapacitor material [56]. The AC material they used as the electrode was 80 wt % carbon powder, 10 wt % extended graphite, and 10 wt % Teflon (dry) suspension. They have the advantages of high exterior area, cost-effectiveness, accessibility, recognized probe production, and machinery and follow EDLC charge storage mechanism and their capacitance mostly depends on the attainable exterior area towards electrolyte ions. Some other constraints include specific surface area (SSA), pore size, assembly, pore size delivery, surface functionality, as well as electrical behavior to alter their performance. Carbon-based ingredients—for example, AC [57], CAGs [58], CNTs [59], and GN [60]—help high ion dispersion also donate to a higher SC of carbon-based composite substances for conducting polymers (CPs)/MO_x_ with excellent cyclic stability [61].

**Figure 5:**
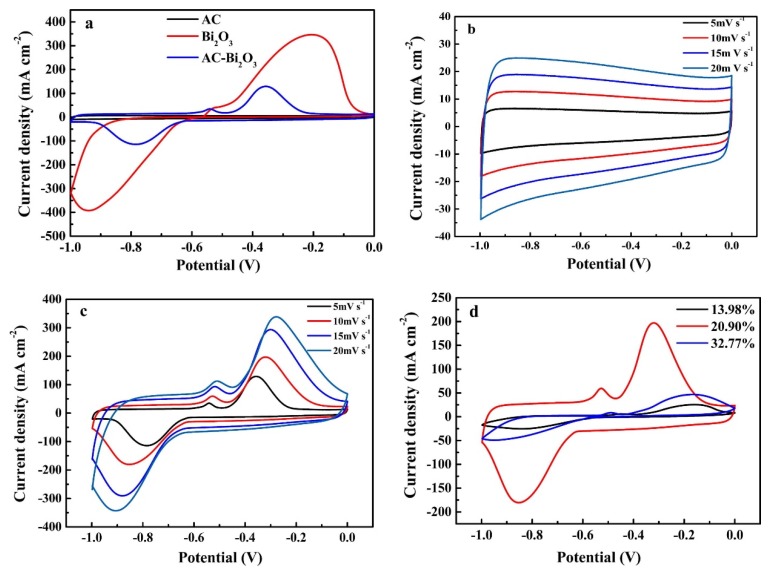
Cyclic voltammogram curves of (**a**) CVs of AC, Bi_2_O_3_ and AC-Bi_2_O_3_; (**b**) CVs of AC at different sweep rates; (**c**) CVs of AC-Bi_2_O_3_ at different sweep rates. (**d**) CVs of different contents AC-Bi_2_O_3_ composite. Reproduced with permission from ref [62].

AC is of great exterior area, excellent electrical possessions, reasonable price due to physical/chemical activation, SA up to 3000 m^2^/g, broad pore size distribution furnishes specific capacitance 100–300 F·g^−1^ in aqueous and <150 F·g^−1^ organic solution. The shape of the CV curve of carbonaceous materials tends to be nearly rectangular (Figure 5) due to the EDLC mechanism (an ideal EDLC should have a rectangular CV and CD curves are straight lines) [62].

Another material is CNTs having exceptional pore construction, virtuous mechanical also thermal steadiness, greater electrical assets, and consistent mesopores that permit a constant charge circulation. CNT has inferior ESR than AC since electrolyte ions may dissociate through the mesoporous system but low SSA (<500 m^2^/g) less than AC which leads to low ED. CNT can be single-walled (SW) or multiwalled (MWCNT) and can be chemically activated with KOH resulting towards improvement within the specific capacitance (*C_s_*) [63].

Apart from AC and CNTs, GN—an outstanding carbonaceous material—is a one particle dense film 2D assembly has good electrical performance, chemical durability, and a wide exterior area (nearly 2630 m^2^/g) [64]. If the whole exterior area of GN is utilized, it can give *C_s_* up to 550 F·g^−1^. GN has a high surface area as together key exteriors of the graphene sheet are eagerly available for electrolyte ions. Reduced graphene sheets showed ED 85.6 Wh/kg on ambient temperature 136 Wh/kg at 1 A·g^−1^ [50] which is equivalent to Ni metal hydride battery. There are different methods have been reported by scientists to synthesize GN comprises chemical vapor deposition, arc discharge method, micromechanical exfoliation, unzipping of CNT, electrochemical, and intercalation approaches; but, in all methods, agglomeration and restacking of GN is a problem for mass production which is the only concern for its commercial use in many applications.

Also, carbon black (CB) is the most commonly used conductive additive because of its great electrical conductivity and small price, but it has a drawback that the carbon particles are not able to form carbon to carbon connected conductive networks sometimes. It results in a reduction in activity over a few thousand cycles. Therefore, other carbon materials—such as acetylene black, AC, GN, and MWCNTs—can be used instead of CB to increase stability [65]. 

#### 3.1.1. Carbon Nanotubes 

**Figure 6:**
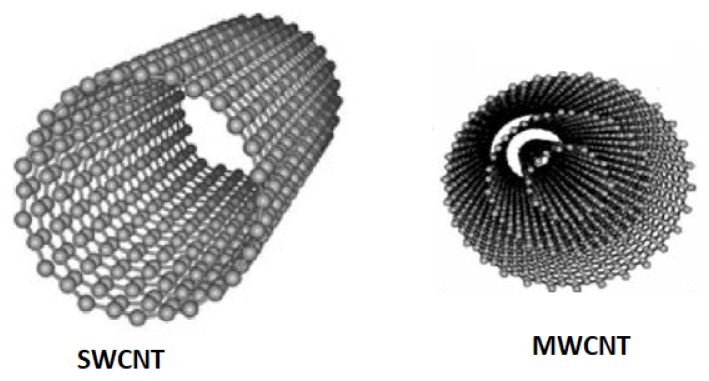
Structure of CNT (SWCNT and MWCNT) carbon-based nanomaterials.

CNT (Figure 6), a carbon-based material, due to their greater physical properties—for example, high chemical steadiness, feature proportion, mechanical assets and activated surface area in addition to extraordinary electrical assets, and nano-dimensions—has obtained exceptional attention owing to its exciting features. The important purpose of all the fascination nanotechnology has gained is mainly owing to the considerable variation in the feature of material while associated with its atomic and massive state [48]. An exceptional pore structure for charge storing but relatively high materials cost and limitation to additional expanding the active exterior area of the CNTs restrict the commercial application of CNTs based SCs. The high aspect ratio of CNTs helps to create CNT fiber that gives about 10 GPa highly tensile continuous fiber [66]. Highly oxidized SWCNTs of electrical conductivity in the range 100–1000 S·cm^−1^ have been reported to have high performance in supercapacitors and Li-ion batteries as electrochemical conductors [67]. More excellent conductivity and improved charge transmission cylinders of CNTs deliver the greatest encouraging element toward energy-saving purposes. In modern generations, CNTs have been considered as valuable power conductor substances owing to their enhanced electrical performance in addition to the highly-available exterior area.

#### 3.1.2. Graphene, Graphene Oxide, and Reduced Graphene Oxide

Graphene (GN) is a carbon-based material (2D form of graphite) having a high exterior area (2675 m^2^·g^−1^) great mechanical strength, greater electrical conductivity (6000 S·cm^−1^), chemical steadiness was first discovered in 2004, and since then has gained significant attention and use in a variety of applications. It has had great influence under the area of science and technology and is a potential candidate for EDLC electrodes [68]. It comprises a single film, while graphite contains a few films of GN loaded composed through van der Waals interaction and π–π stacking. Agglomeration and stacking of layers reduce its effective exterior area.

Graphene oxide (GO) is a solitary film of sp^2^ hybridized carbon particles, arranged in a honeycomb matrix structure, having oxygen as functional assemblies on its basal planes, boundaries and may be developed through exfoliation of GN. GO has been found to be promising carbon material in forming functional nanocomposites due to oxygen functional groups and GO’s compatibility with polymers [69]. 

**Figure 7:**
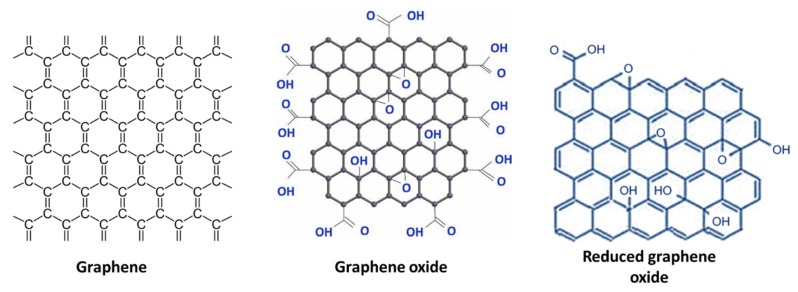
Chemical assembly of GN, GO, and rGO.

From a sustainability viewpoint, the hunt of renewable carbon reservoir and research of economical yet straightforward assembly methods are of selective interest in producing GN and their carbon-based analogs within existing power storage purposes. Although, the standard economic policies acquired so far for GN and GN-like substances construction yet cannot compete with the generation of AC [70]. Chemical assembly of GN, GO, and reduced graphene oxide (rGO) are exposed in Figure 7 [71].

Usually, incorporation of GN through chemical oxidation generates GO by entering oxygenated functional assemblies (carboxyl, hydroxyl, and epoxy) in among the carbon films of GN employing oxidants through Hummer’s protocol [72,73,74]. Therefore, the exterior of GO tolerates negative controls owing to his oxygenated practical combinations. The oxygenated efficient assemblies of GO are useful into combining GO by another active kind such these practical clusters may work as an effective fastening position. In contrast to GN, GO is non-conductive owing to the sp^3^ hybridization of carbon particles, in addition, the functionalization of GO creates holes at the carbon basal extension owing to the change into the sp^2^ hybridized carbon arrangement of the GN layers. To recover the conductivity of GO, deoxygenating or conversion of GO should be brought out.

GN may be modified by chemical reactions plus functionalization towards improving the dispersibility and method ability to improve the synergy through organic polymers owing to the hydrophilic oxygenated functional combinations existing in GO. A quasi-1D single-layer GN nanoribbon (120 nm in width and ~100 µm in length) microelectrode made-up through mechanical exfoliation of graphite, trailed with electron beam lithography method, and oxygen plasma engraving behavior has been investigated as the consequence of chemical functional groups and physical internment at the electrochemical activity [75]. During an electrochemical reaction, the arrangement and localization of electrolyte ions and structuring close to the GN interface can affect the properties at the interface [76]. Application of thermally flaked graphene nanosheets as supercapacitor probe substances has been described to provide the highest *C_s_* of 117 F·g^−1^ in acidic media. Toward supercapacitors composed of chemically transformed GN, a *C_s_* of 135 F·g^−1^ in alkaline media has been described. A novel 3D ultrathin porous carbon nanosheet has been prepared from biomass by hydrothermal method followed by carbonization and tested for SC application that showed ED (79.4 Wh/kg) and PD (5.1 kW/kg), and imposing cyclic constancy by 94.6% after 5000 charge–discharge (CD) methods [77]. 

Reduced graphene oxide (rGO) is an extremely conducting kind of GN may be found either by the chemical reduction of GO by some reducing agent like NaBH_4_, hydrazine, NaOH, Na_2_CO_3_, and L-ascorbic acid or by electrochemical reduction employing CV or chronoamperometry methods on negative voltage. During the reduction GO revives the graphitic arrangement of carbon particles and separates the oxygenated functional combinations at the exterior and sides of GO, so improving the electrical performance of rGO [78]. 

#### 3.1.3. Other Carbon Materials

Carbon quantum dots are sp^2^ graphitic structure and nano-sized carbon particles with oxidized functionalities and semiconducting properties. The main attraction of using carbon dots in nanocomposites can increase the transport of ions in the CD method, which results in profligate redox reactions and high *C_s_* [79]. CAGs having a high electrochemical exterior area carbon fiber materials are entrenched into an incessant system of CAG towards an arrangement of a clear but porous column suggestively improved the exterior area of carbon fiber materials, also later the improved electrochemical activity has been reported [80]. 

### 3.2. Polymer Materials 

Polymers can be natural or synthetic and made of many repeating monomers units united together to form a long chain. To meet the demands of everyday life, synthetic polymers have been playing an important role from the past century. Natural polymers include proteins, enzymes, DNA, in addition, few cases of synthesized polymers are polyethylene, polyester (PE), polyvinyl chloride (PVC), etc., within the arrangement of plastic fibers and the products with exceptional properties and substituting metal counterparts in every industry with cost-effectiveness [81].

Conducting polymers (CPs) are organic materials where the redox method is applied to stock and release charge, therefore it shows an example of pseudocapacitance [82]. If oxidation occurs ions are moved toward the polymer backbone. During reduction, ions are unrestricted posterior within the electrolyte media. These redox reactions in CPs cause mechanical stress results in limiting stability as of many charge discharges cycles which hinders their application as SC materials. Examples of CPs include PANI [83], polypyrrole (PPy) [84], polythiophene (PTh) [85], etc. PANI has better conductivity, facile synthesis, outstanding capacity, and lower cost. However, owing to repetitive series (CD procedure), this causes puffiness and shrinkage that lead to quick degradation. 

Polymers having conducting behavior are of prime interest for electrochemical applications such as solar cells [86], sensors [87], energy storage devices [88], and electronics equipment. The electrochemical energy storage devices like supercapacitors, Li-polymer batteries have been using CPs as high-efficiency electrodes that show reversible redox reactions in electrolytes. Insertion of carbon materials with CPs results in an increase in the stability of the nanostructured materials. Furthermore, in contrast to inorganic resources, the outstanding elasticity of CP also aids in building a supple probe, as well as the flexible tools for wearable electric arrangements [89]. CPs have gained considerable interest and attention due to their conductivity, redox behavior to investigate their necessary substances for exceptional purposes, for example, energy-storing and transformation tools, electrochromic arrays, batteries, membranes, anti-destructive films, etc. [90]. 

CPs, for example, poly(3,4-ethylene dioxythiophene) (PEDOT) [91], PANI [92], PPy [93], and PTh [94]—owing to their outstanding characteristics such as facile construction, affordability, good electrical performance, chemical endurance, excellent environmental resistance, mechanical adaptability, redox activity, and biocompatibility—are attracting considerable interest and are valuable for numerous purposes, with electrochemical energy storage tools [88], actuators, and neural boundaries. These CPs have delocalized π-electron arrangement, available redox conditions, and manageable physical assets that make these conjugated polymers perfect applicants for developing energy storage materials and device fabrications. CPs can accumulation charges by the method of doping. There are two kinds of doping: p-doping (oxidative), which includes the discharge of π-electrons; and n-doping, which gives clear negative charges. PEDOT may support mutually p-plus n-doping, whereas PANI and PPy support p-doping being their n-doping voltage is significantly less comparable to the standard electrolyte reduction potential.

To build effective CP electrodes/probe, it is usually advisable to store a compact CP film at the electrode because its activity is hugely reliant upon this layer’s depth; for example, the areal ED/PD (Wh/cm^2^, W/cm^2^) or activating drive of a CP probe is equivalent to its thickness [95]. It has been noticed that the initiation of MWCNTs within the polymeric patterns increases the specific exterior area and recovers the electrical performance and mechanical characteristics [96].

**Figure 8:**
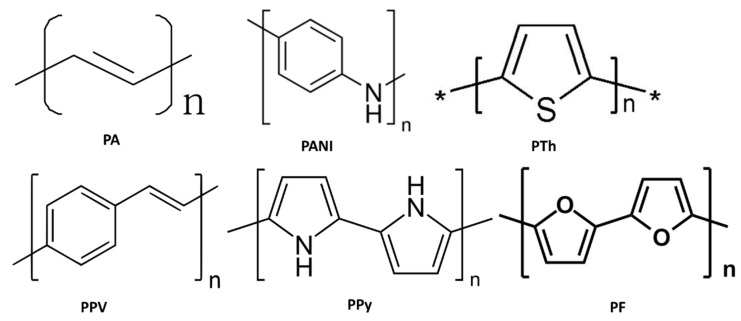
Chemical construction of common ICPs with PA, PANI, PTh, PPV, PPy, and PF.

PANI having cost-effective, facile construction, great flexibility, and high *C_s_* plays a significant part in the energy accommodation and regeneration patterns. The only limitation is the poor cycle life or durability owing to growing and shortening throughout the doping and dedoping method which drives to the architectural destruction of PANI electrode results in poor charge/discharge capability that can be improved by the addition of carbonaceous materials and metallic compounds, and the composite proved to be potential electrode material [92]. Therefore, the tremendous mechanical robustness of carbon-based substances—for example, GN—is utilized continuously to succeed the mechanical degeneration of CPs and head to high cycling endurance, which is necessary for SCs [78]. These nanomaterials in PANI may decrease the dissipation time, improve the electroactive zones, and notably improve the capacitive activity of nanocomposites that can result in enhanced stable specific capacitance due to synergistic effects [79]. Including the discovery of conductive polyacetylene (PA) during the 1970s, further intrinsically conducting polymers (ICPs) have drawn notable recognition from both science and engineering associations. ICPs usually involve PA, PANI, PPy, PTh, poly(phenylenevinylene) (PPV), polyfuran (PF), etc. Their constructions are illustrated in Figure 8.

Investigators are yet concentrating on the advanced substances like carbon-based, metal oxide and CPs or a composite of these. Carbon materials are considered for properties such as great specific area and normal pore size dispersion although their capacitances and energy densities are yet small. With CPs, the challenge is the short lifetime owing to puffiness and dwindling all through CD besides high specific capacitances. Metal oxide possesses high SSA and favor the diffusion of ions. Metal complexes have lower crystallinity and higher SC due to accessibility of chemically active hanging components that can participate in redox cycles [97]. 

### 3.3. Carbon Polymer Nanocomposites 

Composites materials consists of two or more constituents that may have superior properties than the individual constituents. The reinforcement material typically transmits the distinctive characteristics to the matrix where the matrix and strengthening substantial accompaniment each other for their excellent synergy. If the sizes of materials are in the nanoscale then they are known as ‘nanocomposites’. Nanocomposite materials are making a great impact on research and technology globally. The nanofiller reinforced composites possess toughness with stiffness as nanofillers offers an ultrahigh interfacial area with greater thermal and oxidative stability arises from the high surface to volume ratio. Nanocomposites can be classified into three kinds: metal, ceramic, and the polymeric matrix. Our primary interest in this review is polymeric matrix nanocomposites [48]. 

Polymer composites are composite materials that have been employed towards a diversity of purposes and receiving attention in daily routine. Carbon polymer composites have been formed by using carbonaceous materials as a filler or reinforcement material in the polymer matrix. The incorporation of CP with carbon nanomaterials results in nanocomposite electrodes with high ED and PD simultaneously, owing to the combination of pseudocapacitance and EDLC [89]. To form carbon polymer nanocomposite, proper selection of fillers assists in enhancing the electrical possessions of the polymer nanocomposites where CB, GN, and CNT are conducting fillers whose shape and size and amount of loading change the properties of the nanocomposite [98]. 

The discovery of CNT and GN has boomed the field and their incorporation inside the polymer matrix results in enhancement of properties and is rapidly expanding. Related to a various array of nanofillers, CNTs have been developed as the common encouraging nanofiller for polymer composites owing to their exceptional mechanical and electrical assets [99]. 

AC is a widely known electrode material, but transport of ions is slow through the micropores. The porous structure can expedite ion transportation by providing a pathway to ions and therefore a 3D ordered macroporous (3DOM) carbons by a classified porous construction may enable ion conveyance, showing high activity as SC probes have been reported to exhibit. CP-macroporous carbon composite probe substances were developed through deposition of a thin film of PANI at the exterior of 3DOM carbon shows *C_s_* of 1490 F·g^−1^ was perceived at the deposited PANI into the compound probe with excellent rate display and cycle strength were obtained at the complex conductor [100]. 

**Figure 9:**
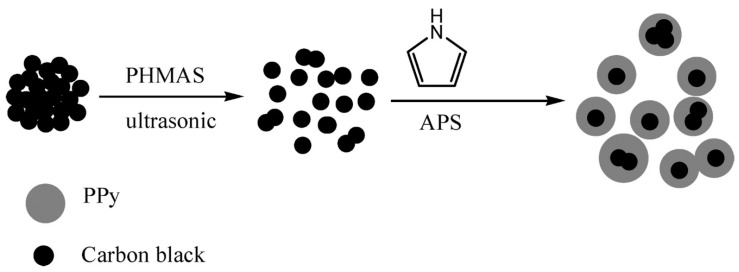
Diagram drawing of the synthesis procedure of the core–shell CB/PPy nanomaterial. Reproduced with permission from [51].

Numerous studies have reported on the PPy/carbon compound being employed for probe substances, for example, PPy/AC [101], PPy/MWCNT [102], PPy/carbon foam [103], PPy/ carbon fiber [104], composites. CB/PPy composites are synthesized by the simple chemical oxidative polymerization of pyrrole by the exteriors of the CB nanoparticles—through poly(2-hydroxy-3-(methacryloyloxy) propane-1-sulfonate) (PHMAS), for example—mutually the surfactant and the dopant. The synthesis process of the core–shell CB/PPy nanocomposite shown in Figure 9 [51].

The nanocomposites of CPs including MO_x_, CNTs, or GN were incorporated mostly through in situ polymerization within suspensions comprising monomers. Including the extension of an oxidant solution comprising ammonium peroxydisulfate [(NH_4_)_2_S_2_O_8_], polymerization of aniline at the exterior of CNTs happened to develop CNTs-PANI nanocomposite. GN-PANI nanocomposite coating with layered construction was achieved by the percolation of an aqueous distribution containing positively charged PANI nanofibers and negatively charged chemically changed GN films which produces a steady composite diffusion by electrostatic synergy due to the support of the sonication process [105]. 

**Figure 10:**
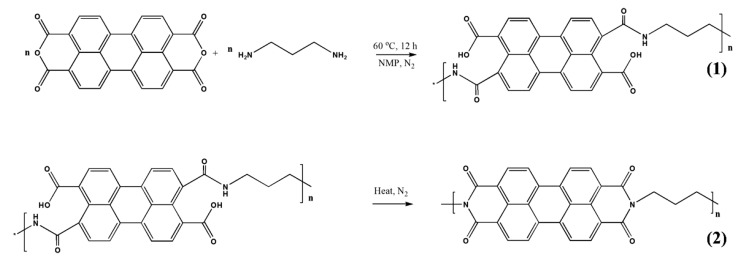
Representation of the synthesis of the PI/MWCNT nanocomposite. Reproduced with permission from [71].

A new polyimide (PI)/ MWCNT nanocomposite by a two-step imidization was synthesized and used as a negative electrode substance for an organic Na-ion battery. A two-step polycondensation reaction made the PI/MWCNT nanocomposite according to the synthetic track presented in Figure 10. Besides its exceptional mechanical resistance, MWCNT works as a conductive material under the nanocomposite and promotes quick electron transmission toward the cathode, and so takes full advantage of the exploitation of the working substance [71].

## 4. Applications of Carbon-based Polymer Nanocomposites

The advances in sustainable and renewable energy storage by renewable energy resources are in great demand to compensate for the energy crisis from conventional energy resources. Electrochemical storage of energy is a medium to store energy for later use that includes electrochemical capacitors, batteries, and FCs that are into urgent demand to develop environmentally friendly energy solutions [106]. The scope of nanoscience and nanotechnology has provided nanocomposites materials with superior properties to be used in industrial and technological development for a wide range of devices. Carbon-based polymer nanocomposites provide a wide spectrum of opportunities to produce novel multifunctional materials individually and on hybridization for applications in thermal materials, electromagnetic interface shielding, sensors, and energy storage [107]. 

Carbon materials with AC, CNTs, and GN have been widely investigated for SCs applicability even in common they represent smaller ED owing for the quick ions adsorption response, for producing EDLCs. On another side, metal oxides and CPs may give more leading EDs by Faradic reactions including low cyclic durability and PD associated with EDL based SCs. Consequently connecting these substances within their compound construction and applying various charging devices and potential combined impact among each of their ingredients with great aspect proportion, the huge exterior area, and electrical performance is known to be a perfect explanation to enterprise and advance the activity of SCs [106,108]. 

### 4.1. Applications of Carbon-Based Polymer Nanocomposites (CPNCs) in Fuel Cells

A FC is crucial energy expertise that has been getting growing recognition to giving substitute environmentally concerned and extremely useful power dynamos during the prior two decades, despite modular in structure by truncated chemical and sound polluting. FCs are electrochemical methods that produce electricity continuously provided the fuel (e.g., H_2_) under the appearance of an oxidant, dissimilar batteries holding yields restricted over their sustained chemical power. Furthermore, FC current densities and energy per mass or size are more important compared to different standard power origins. FCs utilise polymer electrolyte membranes (PEMs), for example, PEMFCs and direct methanol fuel cells (DMFCs), such as solution media are the usual encouraging applicants towards low-temperature performances plus mobile and compact applicability [109]. 

FCs are usually categorized based upon the working circumstances (e.g., temperature), construction (e.g., range of the practice and purpose), and the characteristics of the polymer electrolyte in the FC [30,110]. Table 2 summarizes the working and functional characteristics of the five foremost kinds of FCs.

### 4.2. Applications of CPNCs for Li-Ion Battery

Li-ion batteries are among the most encouraging, practical, and virtuous conventional ED methods employed for electrochemical energy storage. The Li-ion battery’s efficacy informs its widespread application in electricity storage, as it shows an inherently great ED and configuration versatility. Generally employed in ‘nomadic’ electrical gadgets as batteries also seems to indicate that they are an essential element in decreasing CO_2_ emissions, (i) as a potential energy source for progressive electric carriers and (ii) as a possible buffer energy storing method for handling the alternative renewable energy sources. Therefore, the product of plants and animals’ usage of Li-ion batteries is supposed to expanding [111]. Currently, the application of Li-ion batteries in conventional electronic tools—as well as research to obtain further practical and reliable batteries—has grown remarkably. Batteries—with their notable characteristics such as better mechanical features, higher performance, and compactness—are needed for the production of handheld electronics to proceed apace with the rapidly-evolving computing ability, bigger screens, and smaller form factors of these devices. Moreover, there is a growing importance for polymer-based batteries to be combined, including elastic, smooth, and micro-scale electronics. There has been a notable improvement in the problems correlated with these batteries. The exploitation of combustible organic diluents as a conducting solution, growth of Li dendrites, and significant volume variation as an outcome of weak architectural durability are between the obstacles connected by Li-ion batteries [112]. 

CPs are encouraging ingredients towards organic–inorganic composites for use in Li-ion batteries, owing to their unique properties that include good coulombic proficiency and electrical performance. This encourages the design of a battery’s periodical use with slight degeneration. CPs show various benefits, like excellent processability, lower price, acceptable molecular change, and light weight when employed as electrodes. Though poor endurance while driving and low conductivity within degraded state hinder their additional purposes during Li-ion batteries [113]. CPs can be applied as both anodic and cathodic substances, however, they are often employed as cathodes into Li-ion batteries. Various CPs shows considerably diverse EDs, for example, PPy-based probes have EDs of around 10–50 W·h·kg^−1^ and PDs about 5–25 kW·kg^−1^; PANI-based probes give EDs around 50–200 W·h·kg^−1^ and PDs of 5–50 kW·kg^−1^; PTh-based probes give EDs of 20–100 W h·kg^−1^ and PDs of 5–50 kW·kg^−1^ [114,115]. Cheng and co-workers employed directed PANI nanotubes incorporated by HClO_4_ amalgamated as anode probe substances showed better activity compared to the commercial PANI particles into Li-ion batteries [116]. The Li-PANI battery achieved a highly efficient discharge range of 75.7 mAh·g^−1^ and managed a 95.5% of the most potent discharge capacity subsequent to 80 runs. However, the PD is comparatively small, the durability of organic substances presents a severe obstacle [117]. 

Liu and co-workers [118] proposed the development of the design of complex nanomaterials for energy storage. Precisely, exceptional Li-battery chemistries require a standard shift over electrodes which incorporate Li to receive elements based on progress or alloying devices, where the expanded ability is usually followed by extreme volumetric variations, significant bond cleavage, poor electronic/ionic conductivity, and variable electrode/electrolyte interphase. 

**Figure 11:**
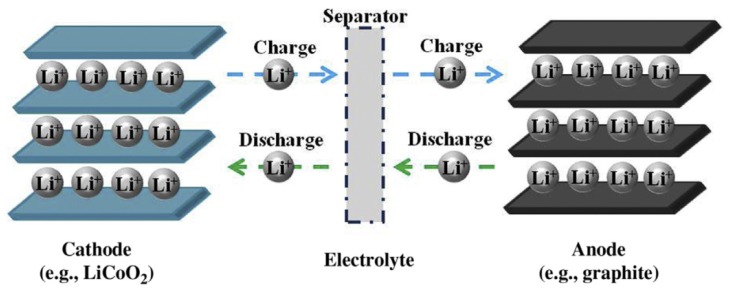
Diagram of the CD procedure in a Li-ion battery. Reproduced with permission from [10].

Among the benefits of high functioning voltage, great storing ability, lowering poisonousness, and continued running period, LIBs have grown to be the most influential and extensively employed rechargeable batteries. The electroactive organic efficient clusters within ICPs have more active redox reaction kinetics compare to common inorganic LIB probe substances. Lately, carbon-based composite probes employing CNTs and GN have been developed that have established the capability and rate recitals of the Li-ion battery. Besides, CPs, that are necessarily PPy and PANI is further used toward the Li-ion battery due to their electrical determination and chemical resistance. Here, we address the purpose of the carbon-based CP composites in the Li-ion battery with their synthesis approaches, structural modification, and electrochemical activity [10]. The CD response of Li-ion batteries depends on the “rocking-chair” notion [119], which is publicized in Figure 11.

**Figure 12:**
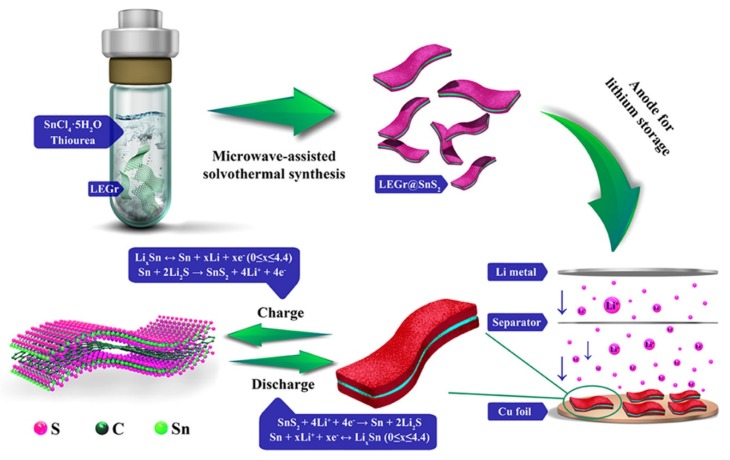
Schematic of the synthesis method and applications of LEGr@SnS_2_ heterojunctions. Reproduced with permission from [121].

Sarang et al. [120] examined the n-type redox reaction with poly-fluorene-alt-naphthalene diimide (PFNDI) by way of an organic battery conductor with the maximum capacity noted towards the compound probe being 39.8 mAh/g at 0.5 C, with an n-doping near of 1.6 Li^+^ ions/reappearance component. GN@SnS_2_ heterojunction nano compound is produced by a microwave-assisted solvothermal method at liquid-phase exfoliated GN (LEGr) [121]. The storing capacity remained 664 mAh·g^−1^ over 200 subsequent runs on 300 mA·g^−1^ current density. The modification procedure and purposes of LEGr@SnS_2_ amalgams are shown in Figure 12.

Based on prior studies, nanotechnology has been giving exceptional resolutions on battery investigation. Among several intricate nanostructure studies, investigators have grown nearer to nailing the enigmas of next-generation battery chemistries. Therefore, it is an excellent opportunity to evaluate development done up until now to extrapolate what might occur in the near future. This report strives to review the critical function of nanotechnology in superior battery methods, highlighting illustrative patterns of Si and Li metal anodes, S cathodes, and composite solid electrolytes. We next address nanomaterials for supercapacitor applications in more detail.

### 4.3. Applications of CPNCs for Supercapacitors 

Due to their high ED and PD, SCs show prodigious latent as high-performance power foundations towards developed machinery. SCs, ultracapacitors or electrochemical capacitors (ECs), are energy-storing tools which store the energy as a charge at the probe exterior or sub-surface film, preferably within the bulk substance as under batteries. As CD transpirs at the facade, it does not cause radical structural reforms against electroactive substances, SCs hold great cycling facility. Owing to those novel characteristics, SCs are perceived as one of the maximum encouraging energy-storing designs [122]. 

There are two kinds of SCs: EDLCs and pseudocapacitors. In EDLCs, the energy is saved electrostatically on the probe and conducting solution edge into the double layer, although the pseudocapacitor charge storage happens through quick redox reactions at the electrode exterior. Here there are three significant kinds of conductor substances for SCs: carbon-based substances, MO_x_/hydroxides, and CPs [123]. EDLCs are only at the exterior part of the carbon-based substances toward a storage charge, hence, they usually show more leading power production and strong cycling capacity. Though, EDLCs have more profound ED values than pseudocapacitors as they include active redox substances to store charge both at the exterior and at the sub-surface film [124]. While carbon-based substances, MO_x_/hydroxides, and CPs are the usual electroactive resources for SCs, every kind of matter has its strengths and limitations, such as carbon-based elements having excellent PD and long life cycle, while their short *C_s_* (mostly double layer capacitance) restricts their use in high ED tools. 

MO_x_/hydroxides maintain pseudocapacitance, as well as double-layer capacitance, and also have a broad charge/discharge voltage scale; however, they have a comparatively low surface area and very poor cycle period. CPs have the benefits of significant capacitance, excellent conductivity, low cost, and efficiency of modification, though they have comparatively short mechanical durability and run time [125]. Joining the unexpected benefits of these nano-scale different capacitive elements to develop nanocomposite electroactive substances is a critical path to regulate, improve, and augment the compositions and characteristics of probe substances for their SC activity. The attributes of nanocomposite electrodes depend not just in the unique ingredients employed but also on the morphology and the interfacial aspects. 

**Figure 13:**
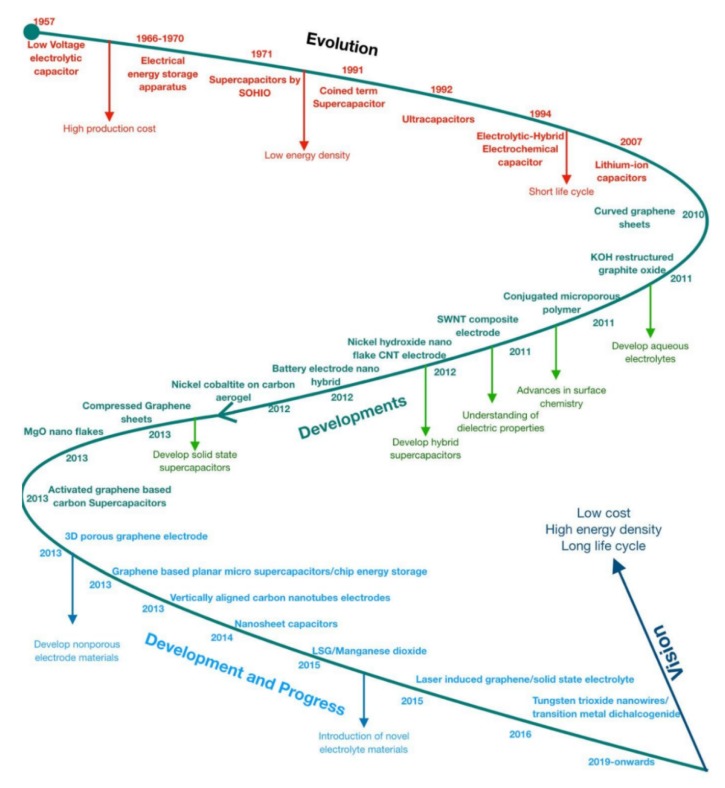
Roadmap of the evolution, progress, and developments of supercapacitors. Reproduced with permission from [126].

Lately, significant works have been allocated to manifest every class of nanocomposite capacitive element; for example, different metal oxides, CPs, CNTs combined with CPs, and GN merged by metal oxides or CPs. Study and invention of nanocomposite electroactive substances for supercapacitors applicability require the attention of several circumstances, for example, material choice, construction techniques, modification method parameters, interfacial properties, electrical performance, nanocrystalline dimension, exterior area, etc. Although an important journey has been completed to improve nanocomposite electroactive elements for SC purposes, there are still many hurdles to be overcome [18]. The stage-wise evolution, research, and development of supercapacitors (and their properties and limitations) are shown in Figure 13 [126].

Flexible solid-state SCs (FSSCs) head within energy-storing expertise also has drawn widespread recognition due to important novel discoveries toward new wearable microelectronics. Remarkable possible useful purposes—for example, the improvement of piezoelectric, outline, retention, reconstruction, electrochromic, and combined sensor-SCs—are further explained [127]. 

CPs, for example—PANI, PPy, and PEDOT—are additional kinds of pseudocapacitive substances by the excessive potential to deliver outstanding *C_s_* [128,129]. Xiao et al. [130] developed a distinct rGO/PANI/rGO double-decker composition nanohybrid paper and also investigated its potential as a probe to SSCs. Primarily, the self-supporting GN paper was developed through a print method and sparkling delamination process and demonstrated excellent electrical performance (340 S·cm^−2^), light weight, and superior mechanical characteristics. Consequently, PANI was electro polymerised at GN paper by continuous deposition of a slight GN film with a concavity layer to develop a sandwich-structured GN/PANI/GN paper. Interestingly, this novel strategy developed the energy storage ability, the rate execution and cycling durability of the probe. Therefore, the as-achieved SSC showed an exemplary capacitance around 120 mF·cm^−2^, which was affirmed at 62% following an improvement in current density on or after 0.1 to 10 mA·cm^−2^, including an ED about 5.4 mW h·cm^−3^.

Current localities are remaining existing at the novel technology that allowed new substances and techniques to energy storage tools. Especially, carbon-based nanomaterials such as GN, carbon nanosheets, CNTs, AC, CAGs, MO_x_, CPs, and polymer amalgams have played a vital role within extremely effective supercapacitors [131]. Carbon-based electroactive [132] and PANI nanocomposite material are employed in SC applications [133]. Among the different construction and heteroatom doping, the nitrogen-doped AC matter delivered a maximum capacitance condition around 268 F·g^−1^ under symmetric SC agent in the acidic media, in addition, 226 F·g^−1^ under the organic environment with 3 V potential windows. The high ED of 60.3 Wh·kg^−1^ received within NAC based SC equipment, intimating the excellent potential for technical employment within the energy storage area [134]. GO/PANI nanomaterial, including PANI nanoparticles, consistently covered above GO films has been strongly developed by the support of CO_2_. GO/PANI nanomaterial by aniline absorption on 0.1 M shows high *C_s_* (425 F·g^−1^) on a current density of 0.2 A·g^−1^. The unique electrochemical capacitance and cycle durability owe to the combined impact among the small nanosized PANI nanocomposites and GO by high specific surface area [135]. 

**Figure 14:**
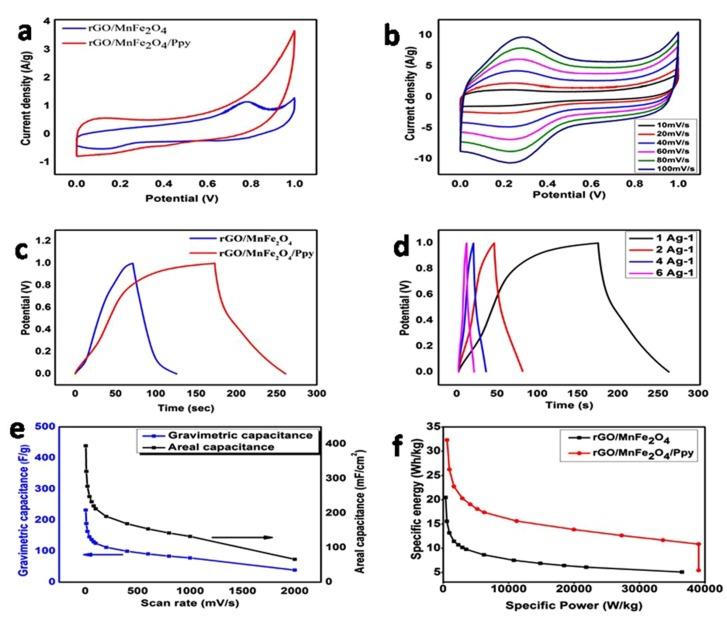
(**a**) CV curves at 5 mV·s^−1^. (**b**) CV curves for rGO/MnFe_2_O_4_/Ppy nanomaterial on various sweep rates. (**c**) CD curves on 1 A·g^−1^. (**d**) CD curves for rGO/MnFe_2_O_4_/Ppy on dissimilar current density. (**e**) Gravimetric and areal capacitance of rGO/MnFe_2_O_4_/Ppy on dissimilar sweep rates (5–2000 mV·s^−1^) (**f**) Ragon designs of rGO/MnFe_2_O_4_ plus rGO/MnFe_2_O_4_/Ppy on dissimilar sweep rates (5–2000 mV·s^−1^). Reproduced with permission from [106].

A paired composite of GN by incorporating iron oxide (rGO/MeFe_2_O_4_) (Me = Mn, Ni) was manufactured employing a novel single-step method by NaOH employing as a coprecipitation and GO proton-rich component. The rGO/MnFe_2_O_4_ compound probe revealed gravimetric capacitance about 147 F·g^−1^ including oxidative capacitance around 232 mF·cm^−2^ on a sweep rate around 5 mV·s^−1^. The ternary GN/metal-doped iron oxide/PPy (rGO/MnFe_2_O_4_/Ppy) compound probe exhibited expressively increased gravimetric capacitance and oxidative capacitance of about 232 F·g^−1^ and 395 mF·cm^−2^, correspondingly showing the combined effect of PPy additives and the corresponding studies shown in Figure 14 [106].

Lately, amalgams of polymers plus nanofillers, for example, carbon-based substances have been favorably accepted as probes toward enhancing the activity of SCs applying the high synergistic impact. Biswas et al. [136] integrated GN/PPy composite material presenting gravimetric capacitance about 165 F·g^−1^ on 1 A·g^−1^ current density contained within two conductor’s arrangements though applying 1 M NaCl media. Parl et al. [137] adopted graphite/PPy compound towards SC electrodes exhibiting gravimetric capacitance around 400 F·g^−1^ held with three probes arrangement. With the aim of obtaining superior SCs activity, the idea of the ternary operation with connecting these three segments has been introduced [138]. The synthesized ternary PPy/GO/ZnO SC electrodes by calculated its gravimetric capacitance within two probes arrangement to be 94.6 F·g^−1^ on 1 A·g^−1^ by CD arcs. Furthermore, Lim et al. [139] described the ternary PPy/GN/nano MnO_x_ complex, the gravimetric capacitance of manufactured compound was 320.6 F·g^−1^ on 1 mV·s^−1^ that was abundant advanced compare to the PPy/GN and straight PPy carrying gravimetric capacitance of 255.1 F·g^−1^ and 118.4 F·g^−1^, respectively. Xiong and co-workers [140] applied three probes method to estimate gravimetric capacitance of ternary cobalt ferrite/GN/PANI nanomaterials, that conferred gravimetric capacitance of about 1133.3 F·g^−1^ on the sweep rate at 1 mV·s^−1^. These investigations show that the composition of multi-ingredient compound terminals to SCs is a useful and encouraging path that can suggestively advance the activity of SCs. 

The combination of PPy/CNT has been employed as an assuring pseudocapacitive cathode toward non-aqueous LIC purposes. The PPy gives high pseudocapacitance through the doping/undoping effect and the CNT incorporation significantly improves electrical performance. The as-developed composite gives exceptional capacities and steadiness (98.7 mAh·g^−1^ on 0.1 A·g^−1^ plus hold 89.7% following runs on 3 A·g^−1^ for 1000 cycles within Li-half cell), that exceeds porous carbon negatives within existing LICs. Moreover, while joined by Fe_3_O_4_@C positive electrode, the as-developed LICs manifests a greater ED around 101.0 Wh·kg^−1^ on 2709 Wh·kg^−1^, and yet preserves 70 Wh·kg^−1^ through improved PD of 17,186 W·kg^−1^ [141]. 

Recently, carbon nanocomposites (particularly, CNTs plus GN) have been extensively examined as active electrodes in SCs due to their excellent specific exterior area and outstanding electrical and mechanical traits. Current investigation and expansion evidently specify that high-performance SCs can be equipped through electrodes based on vertically-aligned CNTs by unlocked tips, GN sheets with tenable through-thickness π–π stacking connections and/or edge functionalities, and 3D pillared GN-CNT systems. The various materials and their supercapacitor performances are given below in Table 3.

## 5. Conclusions and Future Prospects

This review article compiles the state-of-the-art and novel hurdles in carbon-based polymer nanocomposites regarding high-performance energy storage applications. Focusing on the possible studies to improve the electrocatalytic behavior of these combined multiphase nanocomposites and principally at the connection among the composition and processing of carbon-based polymer nanocomposites in terms of exploiting the electrochemical efficiency are described.

The fast consumption of fossil fuels and raised contamination has led to the expansion of energy transformation and storage tools. Fuel cells, Li-ion batteries, and supercapacitors are the possible candidates in these applications. Here, in the event of a fuel cell film, the addition of nanomaterials over polymer membranes improves the physical characteristics as well the proton conductivity, and activity of the membranes means that the nanomaterial does not act as a block to proton movement of the anode to the cathode. This can be accomplished by employing proper incorporated and developing the degree of distribution. Notwithstanding lots of studies which have previously been conducted herein area, up to now, no invention has been completed, and are much more necessary to be fulfilled before the edge use.

Currently, CNTs are used as a substitute to graphite-based anodes. CNT-based anodes with tiny advances in Li storage potential and cyclability associated with graphite-based anodes make it challenging to practice them for substituting graphite-based materials in Li-ion substance. It seems that the numerous encouraging efforts toward developing different energy storage devices may originate from the incorporation of CNTs with another composite with intricate nanostructure schemes. CPNCs as an electrode substance have also been studied. Additional investigations are needed to develop their long-term durability. Mostly, carbon-based materials are being accepted as for supercapacitor electrodes.

GN-based substances are admittedly one of the impressive materials with high potential within the active area of energy storage devices. It has shown great specific capacitance together with polymeric nanocomposite. In practice, GN has been considered the perfect supercapacitor electrode substance due to its vast exterior area, notably high electrical conductivity, and high mechanical robustness. Traditionally, however, extensive works are yet required to apply this potential material into an extremely effective element.

Although the progress has significantly delivered more reliable storage abilities and achievement, tailoring, and optimization of substances are yet in a developmental manner where cost-effective and great execution are of the important interests in functional applicability. To generate a succession of high PD and EDs, hybrid technology is one of the exciting features. For that, we require a much perception of surface chemistry among electrode substances and electrolytes to enhance the interfacial synergies beneficial for improved charge transfer. Nanoarchitectures play an important part in building a structure feature synergy among separate elements, for example, CPNCs. Therefore, integrative directed elements require to be intensively examined to achieve an optimized high-performance energy storage materials that can work with the energy crunch we are suffering now.

## Figures and Tables

**Table 1:** Comparison between electric double layer capacitors and pseudocapacitor

EDLC	Pseudocapacitor
Good cyclic stability	Greater specific capacitance
Good power performance	Greater energy density
Formation of the double layer at the interface	No such layer formation
No redox reaction i.e., non-faradaic	Redox reactions, i.e., Faradaic
No mechanism failure	Depends upon redox reactions

**Table 2:** Typical characteristics of various fuel cell systems. Reproduced with permission from [30,110].

Fuel Cells	Anode Feed	Cathode Feed	Working Temp. (°C)	Power Density (mW/cm^2^)	Fuel Efficiency
Alkaline fuel cell	Pure H_2_	O_2_ or air	90–100	100–200	60
PEMFCs	Pure H_2_	O_2_ or air	50–100	350	60
Phosphoric acid fuel cell	Pure H_2_	O_2_ or air	150–200	200	40
Molten carbonate fuel cell	H_2_ or natural gas	O_2_ or air	600–700	100	45–50
Solid oxide fuel cell	Gasoline or natural gas	O_2_ or air	700–1000	240	60

**Table 3:** Various materials and their supercapacitive performance

Substances	Specific Capacitance (C_s_)	Cycling Durability	Ref.
CNT	1.8 mF·cm^−2^ on 1 mA	80%; 1000 runs	[142]
MnO_2_ nanowire/GN	66.1 F·cm^−3^ on 60 mA·cm^−3^	96%; 10,000 runs on 0.12 A·cm^−3^	[143]
AC cloth	161.2 mF·cm^−2^ on 12.5 mA·cm^−2^	104%; 30,000 runs on 12.5 mA·cm^−2^	[144]
AC	153 mF·cm^−2^ on 10 mV·s^−1^	93.4%; 1000 runs on 200 mV·s^−1^	[145]
PANI hydrogel	430 F·g^−1^ on 5 mV·s^−1^	86%; 1000 runs on 7.5 A·g^−1^	[146]
GN	180.40 mF·cm^−2^ on 1 mA·cm^−2^	96.8%; 7500 runs on 8 mA·cm^−2^	[147]
TiO_2_@PANI	775.6 mF·cm^−3^ (28.3 F·g^−1^) on 10 mV·s^−1^	97.2%; 10,000 runs on 100 mV·s^−1^	[147]
NiCo_2_O_4_@CNT/CNT	-	95%; 5000 runs on 50 mV·s^−1^	[148]
MnO_2_/rGO	14 F·cm^−2^ (31.8 F·g^−1^) on 2 mV·s^−1^	100%; 5000 runs on 0.2 mA·cm^−2^	[149]
SnS/S doped GN	2.98 mF·cm^−2^ on 60 mA·cm^−2^	99%; 10,000 runs on 120 mA·m^−2^	[150]
Co_3_O_4_/vertically aligned GN nanosheets	580 F·g^−1^ on 1 A·g^−1^	86.3%; 20,000 runs on 20 A·g^−1^	[151]
MnO_2_-CNT-GN	107 F·g^−1^	-	[152]
N/O-Enhanced carbon cloth	-	116%; 5000 runs on 5 mA·cm^−1^	[153]
MnO_2_/CNT	324 F·g^−1^ on 0.5 A·g^−1^	100%; 5000 runs on 10 A·g^−1^	[154]
GN sheets	11.3 mF·cm^−2^ on 1 mV·s^−1^	-	[155]
PANI-MnO_x_	94.73 mF·cm^−2^ on 0.1 mA·cm^−2^	-	[156]
Graphite/PANI	77.8 mF·cm^−2^ on 0.1 mA·cm^−2^	83%; 10,000 runs on 1 mA·cm^−2^	[157]
KCu_7_S_4_/GN	-	92%; 5000 runs on 0.8 mA·cm^−2^	[158]
Ag/AC	45 mF·cm^−2^ on 0.3 mA·cm^−2^	86%; 1200 runs on 5 mA·cm^−2^	[159]
GN/MWNT	740.9 mF·cm^−2^ on 1 mA·cm^−2^	85%; 20,000 runs on 15 mA·cm^−2^	[160]
SWCNTs	17.5 F·g^−1^ on 2 A·g^−1^	87.5%; 10,000 runs on 5 A·g^−1^	[161]
Au/PANI	26.49 mF·cm^−2^ (67.06 F·cm^−3^) on 0.5 mA·cm^−2^	72.7%; 1000 runs on 200 mV·s^−1^	[162]
GO/MOF	250 mF·cm^−3^ on 6.4 mA·cm^−3^	96.3%; 5000 runs on 50.4 mA·cm^−3^	[163]
rGO/PPy	147.9 F· cm^−3^ on 5 A·cm^−3^	71.7%; 5000 runs on 10 A·cm^−3^	[164]
N-Doped rGO	3.4 mF·cm^−2^ on 20 mA·cm^−2^	98.4%; 2000 runs on 100 mA·cm^−2^	[165]
rGO/MoO_3_	404 F·g^−1^ on 0.5 A·g^−1^	80%; 5000 runs on 2 A·g^−1^	[166]
GN	56.5 F·cm^−3^ on 0.06 A·cm^−3^	-	[167]
b-Ni(OH)_2_/graphene	2570 mF·cm^−2^ on 0.2 A·m^−1^	98.2%; 2000 runs on 0.1 A·m^−2^	[168]
GN/carbon black nanoparticle	144.5 F·g^−1^ on the current density of 0.5 A·g^−1^	-	[169]
GN fibers/MnO_2_ fibers	42.02 mF·cm^−2^ on 0.01 V·s^−1^	92%; 1000 runs on 1 mA·cm^−2^	[170]
GO	130 F·g^−1^ on 5 mV·s^−1^	-	[171]
Cu(OH)_2_//AC	26.4 F·g^−1^ on 4 A·g^−1^	90%; 5000 runs	[172]
ZnCo_2_O_4_//carbon nanofibers	139.2 F·g^−1^ on 2 mV·s^−1^	90%; 3000 runs on 50 mV·s^−1^	[173]
CuMnO_2_-gCN	817.85 F·g^−1^ at 0.025 A·g^−1^	91% up to 1000 cycles	[174]
